# Brain-derived neurotrophic factor as a regulator of systemic and brain energy metabolism and cardiovascular health

**DOI:** 10.1111/j.1749-6632.2012.06525.x

**Published:** 2012-04-30

**Authors:** Sarah M Rothman, Kathleen J Griffioen, Ruiqian Wan, Mark P Mattson

**Affiliations:** Laboratory of Neurosciences, National Institute on Aging Intramural Research Program, National Institutes of HealthBaltimore, Maryland

**Keywords:** autonomic nervous system, brain-derived neurotrophic factor, cognition, diabetes, exercise, neurogenesis, synaptic plasticity

## Abstract

Overweight sedentary individuals are at increased risk for cardiovascular disease, diabetes, and some neurological disorders. Beneficial effects of dietary energy restriction (DER) and exercise on brain structural plasticity and behaviors have been demonstrated in animal models of aging and acute (stroke and trauma) and chronic (Alzheimer's and Parkinson's diseases) neurological disorders. The findings described later, and evolutionary considerations, suggest brain-derived neurotrophic factor (BDNF) plays a critical role in the integration and optimization of behavioral and metabolic responses to environments with limited energy resources and intense competition. In particular, BDNF signaling mediates adaptive responses of the central, autonomic, and peripheral nervous systems from exercise and DER. In the hypothalamus, BDNF inhibits food intake and increases energy expenditure. By promoting synaptic plasticity and neurogenesis in the hippocampus, BDNF mediates exercise- and DER-induced improvements in cognitive function and neuroprotection. DER improves cardiovascular stress adaptation by a mechanism involving enhancement of brainstem cholinergic activity. Collectively, findings reviewed in this paper provide a rationale for targeting BDNF signaling for novel therapeutic interventions in a range of metabolic and neurological disorders.

## Brain-derived neurotrophic factor and food intake

In the central nervous system (CNS), brain-derived neurotrophic factor (BDNF) and its high-affinity receptor TrkB are highly expressed in the hypothalamus, where this neurotrophic factor has major regulatory roles in the control of appetite and metabolism.^1^ Mice that are heterozygous for targeted disruption of BDNF (BDNF^+/−^ mice) show a 50% reduction in BDNF expression in the hypothalamus,^2^ consume 47% more food than wild-type (WT) mice, and are obese.^3^ Obesity in BDNF^+/−^ mice is prevented by restricting food intake to match that of WT counterparts, implying that the loss of BDNF causes hyperphagia, which leads to obesity.^4^ A reduction in the expression of TrkB also leads to obesity in mice.^5^ By six months of age, the average weight of male and female BDNF^+/−^ mice are 44% and 33% increased over WT counterparts, respectively.^3^ This age-related obesity and chronic hyperphagia is accompanied by hyperactivity, which is in contrast to lethargy normally associated with obesity.^3^,^6^

**Figure 1:**
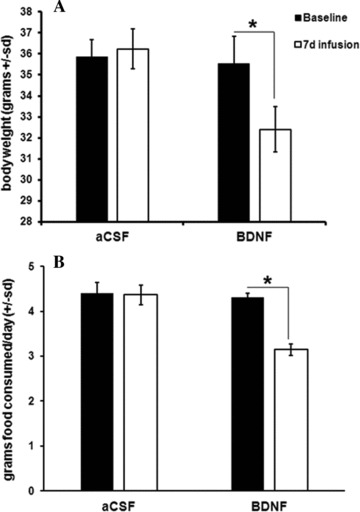
BDNF infusion causes a significant decrease in food consumption and a loss of body weight. ICV infusion of BDNF (1.2 μg over 24 h), causes a significant (*P* = 0.005) decrease in food consumption in WT mice as well as a significant (*P* = 0.01) decrease in body weight. Mice receiving infusion of artificial cerebrospinal fluid (aCSF) do not experience a significant change in either food consumption or body weight.

Diminished BDNF signaling results in hyperphagia and obesity, whereas an increase in BDNF signaling has the opposite effect. Intracerebroventricular (ICV) infusion of BDNF into normal mice results in a significant decrease in food consumption and a loss of body weight (Fig. 1). After seven days of intracerebroventricular (ICV) infusion of BDNF (1.2 μg over 24 h), WT mice experience a highly significant (*P* = 0.005) decrease in food consumption and a significant (*P* = 0.01) decrease in body weight, whereas mice receiving infusion of artificial cerebrospinal fluid do not. This effect of BDNF also occurs in rats.^7^ ICV infusion of BDNF into BDNF^+/−^ mice normalizes food intake, body weight, and activity, further implying a physiological role for BDNF in regulating food intake.

The precise mechanism by which BDNF signaling suppresses appetite and food intake is not entirely known, although several hypotheses have emerged. The regions of the hypothalamus that regulate food intake include the paraventricular, arcuate (ARC), dorsomedial (DMH), and ventromedial nuclei (VMH). BDNF is expressed in some cells in the dorsomedial hypothalamus and at negligible levels in the arcuate; however, it is highly expressed in the VMH. Bilateral lesions of the VMH cause hypherphagia and obesity, implying an important role for this region of the hypothalamus in regulating food intake and energy metabolism.^8^ Leptin, a polypeptide produced by adipocytes, targets neurons in the ARC; leptin positively regulates proopiomelanocortin neurons, which project to the VMH. In the VMH, expression of the melanocortin receptor 4 (MC4R) regulates expression of BDNF; a reduction in MC4R causes a downregulation of BDNF.^5^ Further, administration of an MC4R agonist increased the level of BDNF mRNA in food-deprived mice, implying that MC4R signaling regulates alterations in BDNF in response to food deprivation.^5^ Taken together, these data provide the beginning of a framework by which gut hormones target neurons in the hypothalamus that control expression of BDNF and therefore, food intake and metabolism.

Another region of the brain that may mediate the effects of BDNF signaling on food intake is the brainstem. Specifically, the dorsal vagal complex (DVC) contains insulin and leptin receptors as well as mechanisms that sense glucose levels.^9^ Intraparenchymal infusion of BDNF to the DVC causes dose-dependent anorexia, implying that alterations in BDNF signaling in the DVC, without any alteration in signaling in the hypothalamus, are sufficient to alter feeding and potentially metabolism parameters.^10^

Experimental manipulations of BDNF levels affect feeding and metabolism, and environmental factors such as food deprivation and stress also cause changes in BDNF expression in the brain. Alternate day fasting stimulates the production of BDNF in neurons in different brain regions, including hippocampus, specifically the dentate gyrus.^11^–^13^ It is noteworthy that dietary energy restriction (DER) restores BDNF levels in BDNF^+/−^ mice and also reverses hyperphagia and obesity in those mice.^2^ Conversely, food deprivation has an inhibitory effect on BDNF expression in the VMH of the hypothalamus that is partially reversed by administration of a MC4R antagonist.^5^ In addition, food deprivation causes a reversible decrease in BDNF protein in the DVC, potentially indicating a role for DVC BDNF signaling in mediating metabolic feedback. The reason for the differential, brain region-specific changes in BDNF expression is likely that the neuronal circuits in the different regions are involved in coordinating complex behavioral and neuroendocrine responses to food intake or deprivation. Because food restriction is stressful, it can cause a rise in corticosterone (cortisol in humans), which has been shown to decrease the expression and production of BDNF in the brain. However, the brain stem may respond to food deprivation in a compensatory manner; food deprivation may cause an acute decrease in BDNF signaling, but it is possible that the brain stem upregulates BDNF expression in a compensatory response to a decrease in food intake.

## BDNF regulates peripheral energy metabolism

BDNF regulation of food intake is also coupled to the ability of this neurotrophin to regulate peripheral energy metabolism. Low levels of circulating BDNF are noted in individuals with obesity and type 2 diabetes, implying a role for this neurotrophin in mediating obesity and metabolism.^14^ However, patients with type 2 diabetes that are not obese display decreased levels of plasma BDNF, which could indicate that BDNF regulates obesity and metabolism via different mechanisms.^15^ Mice with BDNF eliminated at birth are hypersensitive to stress, display elevated plasma glucose and insulin levels, and are obese.^6^ Experimental studies show that ICV infusion of BDNF increases peripheral insulin sensitivity in normal rodents^16^ and ameliorates diabetes in mice,^17^ implying that BDNF signaling in the brain, particularly the hypothalamus, regulates peripheral energy metabolism. Further, BDNF^+/−^ mice display a diabetic phenotype, including elevated levels of circulating glucose, insulin, and leptin.^2^

Obesity is associated with changes in the serum levels of leptin, insulin, glucose, and corticosterone/cortisol.^18^ The interplay between these molecules is complex. In normal individuals, insulin is produced in response to increased glucose levels and stimulates glucose uptake by muscle and liver cells; corticosterone induces gluconeogenesis, and leptin production and secretion from adipocytes can be stimulated by insulin; and circulating leptin enters the brain and interacts with neurons in the hypothalamus to suppress appetite. Although the exact mechanisms by which BDNF regulates metabolism are not entirely known, several studies using leptin receptor mutant (*db*/*db*) mice demonstrate an important role of this hormone in BDNF-mediated regulation of metabolism. The *db*/*db* mice are hyperglycemic and insulin resistant, and administration of BDNF normalizes glucose levels and insulin sensitivity.^16^ Further, controlled studies in which both vehicle and BDNF-treated *db*/*db* mice consumed the same amount of food showed that BDNF administration significantly reduces blood glucose concentration, indicating that the effects of BDNF on energy metabolism are independent of its effects on appetite. Further proof of a role for leptin in mediating BDNF regulation of energy metabolism lies in data that shows that BDNF^+/−^ mice display increased leptin levels and leptin resistance.^3^ Conversely, *db*/*db* mice that cannot respond to leptin show reduced BDNF expression in the hippocampus and hypothalamus. Systemic BDNF infusion reduces food intake and blood glucose in *db*/*db* mice.^17^,^19^ Lowering circulating corticosterone levels in *db*/*db* mice via adrenalectomy restores BDNF expression in the hippocampus but not the hypothalamus.^20^

Data show that some of the effects of leptin on energy metabolism may be mediated via changes in body temperature control. *Db*/*db* mice show reduced energy metabolism and lower body temperature and may be unable to activate thermogenesis to maintain body heat when their food intake is restricted.^16^ In fact, administration of BDNF-raised body temperature in food-restricted *db*/*db* mice compared to *ad libitum* mice.^17^

Other models of diabetes in mice have been used to demonstrate a role for BDNF in regulating energy metabolism. Administration of streptozotocin (STZ) damages pancreatic β cells, leading to impaired insulin production and is therefore a model of type I diabetes. In STZ-treated mice, administration of BDNF causes a reduction in food intake but no change in circulating glucose levels, implying that the mechanism by which BDNF regulates food intake is independent of insulin signaling, but the mechanism by which BDNF regulates energy metabolism is insulin dependent.^16^

## Involvement of BDNF in brain-stem control of the cardiovascular system

It is well known that neurotrophic factors such as BDNF are essential for the development of the autonomic nervous system (ANS), particularly in the formation of synaptic connectivity with peripheral targets. For example, BDNF is essential for the survival of arterial baroreceptors during vascular innervation.^21^ However, recent findings indicate that BDNF also plays a major role in ANS control of cardiovascular function in adults. A role for BDNF in ANS regulation of heart rate is suggested by data showing exercise and DER, both of which increase BDNF levels in the CNS,^13^,^22^,^23^ also decrease heart rate and increase heart rate variability.^24^–^26^ The latter effects of exercise and DER are believed to be mediated by a relative increase in parasympathetic activity on the heart. Indeed, Yang *et al.*^27^ showed that BDNF could alter the neurotransmitter release properties of sympathetic neurons innervating cardiac myocytes in culture, resulting in a shift from excitatory to inhibitory cholinergic neurotransmission. The cardioprotective effects of DER may result, in part, from the relative increase in parasympathetic activity.^28^

**Figure 2:**
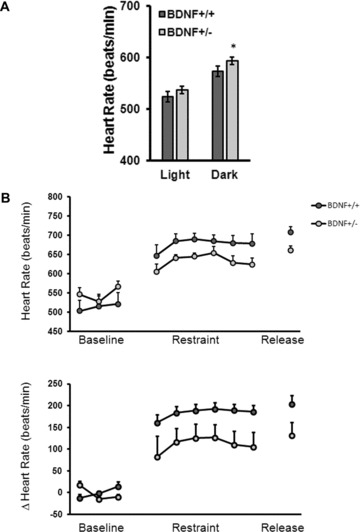
Evidence that BDNF signaling regulates heart rate. (A) BDNF^+/−^ mice have significantly elevated basal heart rates compared to control (BDNF^+/+^) mice during the dark cycle (*P* < 0.05). (B) BDNF^+/−^ mice have attenuated heart rates during a 60-min restraint stress and release. Data points represent 10-min bins (*P* < 0.001). The change in heart rate in BDNF^+/−^ mice is significantly lower than BDNF^+/+^ mice during restraint and release (*P* < 0.001). Data are represented as mean ± SEM.

Studies from our lab further suggest a role for BDNF in regulating parasympathetic and/or sympathetic inputs to the heart. BDNF^+/−^ mice, which exhibit a 50% reduction in BDNF mRNA, have significantly elevated heart rates compared to WT mice (Fig. 2A). Further, when exposed to restraint stress, BDNF^+/−^ mice fail to elevate their heart rate to WT levels (Fig. 2B), indicating an impaired cardiovascular stress response. Interestingly, a recent study revealed that humans with a BDNF polymorphism (Val66Met) that results in decreased activity-dependent BDNF secretion have altered sympathovagal balance leading to sympathetic dominance.^29^ In addition, carriers of this mutation also exhibit attenuated heart rate responses to stress.^30^ Taken together with our data, the latter findings suggest that reduced BDNF expression impairs heart rate stress responses and ANS function.

BDNF regulation of cardiovascular function likely occurs via signaling in central autonomic nuclei of the brain stem. BDNF is expressed in both baroreceptor and chemoafferents in the nodose and petrosal sensory ganglia, which terminate in the brainstem.^21^,^31^ BDNF and TrkB are also produced in central autonomic nuclei of the brain stem,^32^–^35^ as well as in higher cardiovascular control areas such as the hypothalamus, forebrain, and amygdala.^36^,^37^ Local injection of BDNF into the rostral ventrolateral medulla increases blood pressure,^38^ and BDNF acutely raises heart rate and blood pressure when applied to the third ventricle.^39^ Further, BDNF is released in synchrony with baroreceptor activity from baroreceptor afferents onto second-order neurons in the nucleus tractus solitarius (NTS), the primary target of afferent cardiovascular input to the brainstem.^31^,^40^ Injection of BDNF into the NTS of anesthetized rats increases blood pressure, heart rate, and lumbar sympathetic nerve activity.^41^ Conversely, inhibition of tonic BDNF signaling in the NTS with a TrkB receptor antagonist decreases blood pressure and heart rate, indicating that BDNF signaling in the NTS tonically modulates cardiovascular regulation.^41^

## BDNF mediates exercise- and energy restriction–induced neuroplasticity

**Figure 3:**
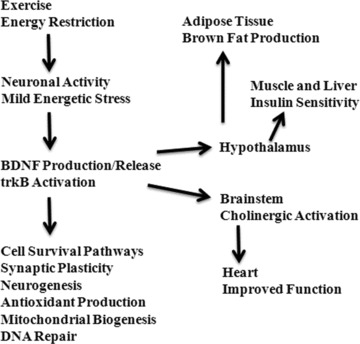
Central roles for BDNF as a mediator of beneficial effects of exercise and dietary energy restriction on neuroplasticity and overall health. Exercise and energy restriction (particular intermittent energy restriction) induce increased activity in neuronal circuits, as well as a mild energetic stress, throughout the nervous system. This mild cellular stress stimulates the production and release of BDNF. BDNF acts on neurons to promote their growth, enhance synaptic plasticity, and increase their resistance to injury and disease. Some of the genes induced in neurons in response to BDNF include those encoding antioxidant enzymes, DNA repair enzymes, and proteins involved in mitochondrial biogenesis. In addition, in the hippocampus, BDNF promotes the production and survival of new neurons from stem cells, and the integration of the new neurons into existing neuronal circuits. Acting in brain regions that control neuroendocrine pathways (e.g., hypothalamus) and the autonomic nervous system (e.g., brain-stem cholinergic neurons), BDNF mediates beneficial effects of exercise and energy restriction on glucose metabolism, body fat composition, and cardiovascular fitness.

The beneficial effects of exercise on health are unequivocal and, as reviewed previously,^42^ include profound effects on the brain. Studies performed primarily in laboratory rodents have revealed highly reproducible effects of exercise, particularly aerobic endurance exercise, on the structure and function of the brain. Compared to their more sedentary counterparts, mice or rats that run voluntarily on running wheels (runners), exhibit increased numbers of dendritic spines (i.e., synapses) in hippocampal neurons, increased neurogenesis, and improved performance in some behavioral tests of cognitive function.^43^–^45^ Mice and rats will typically run between 2 and 15 km in a 24-h period; there is considerable interanimal variability in daily running distance, but less day-to-day variability in daily distance running for individual mice.^46^–^48^ Where examined, there tends to be a graded positive effect of daily exercise intensity and duration on synaptic plasticity and neurogenesis. In addition, the positive effects of exercise on synaptic strength and learning and memory are not immediately apparent, and instead occur over periods of many days to weeks. The purpose of this section is to describe the evidence that BDNF plays a key role as a mediator of the effects of exercise on synaptic plasticity and neurogenesis (Fig. 3). In keeping with the main theme of this paper, we also consider recent evidence that BDNF mediates beneficial effects of intermittent energy restriction (IER) on neuroplasticity and the vulnerability of neurons to injury and disease.^49^,^50^

### Exercise and BDNF

Mice that are runners can perform better in tests of learning and memory, such as the radial arm maze, compared to nonrunners. Interestingly, when mice were provided running wheels for one week and then the running wheels were disabled, they learned the maze fastest when tested one week after cessation of running, but their memory retention was best when tested immediately after the one-week running period.^51^ Hippocampal BDNF levels were elevated immediately after the one-week running period, remained elevated for two weeks after cessation of running and then returned to baseline by three weeks. There is also evidence that prior exercise can prime a BDNF response of hippocampal cells to a subsequent brief period of exercise that would be insufficient to significantly increase BDNF levels in previously sedentary animals.^52^ The priming effect of exercise is maintained for at least two weeks after the cessation of exercise. The underlying molecular mechanism of the priming effect of exercise on BDNF production remains to be determined. In addition to improving performance in learning and memory tasks, running was shown to enhance the ability of mice to distinguish between objects located close to each other on a touch screen (spatial pattern separation), a process dependent upon hippocampal plasticity.^53^ Moreover, a study of elderly human subjects showed that a daily aerobic exercise intervention can improve performance in memory tests, with an associated increase in the size of the hippocampus as measured by structural magnetic resonance imaging methods.^54^

The results of different behavioral tests have revealed that voluntary running and forced treadmill running can differentially affect performance on behavioral tasks. For example, whereas both voluntary and treadmill running improved performance of mice in the water maze, only treadmill running improved performance in a passive avoidance test.^55^ The latter study further showed that both forced and voluntary running upregulated BDNF–TrkB signaling in the hippocampus, whereas only forced exercise increased BDNF signaling in the amygdala. In as much as forced exercise can be considered to be an aversive (or at least an annoying) stressor, it is understandable that it would engage neuronal circuits in the amygdala, a brain region heavily involved in fear-related learning.^56^

In rodents, running wheel exercise induces the expression of BDNF at the transcriptional level in multiple brain regions, and in particularly large amounts in dentate gyrus granule neurons and CA1 neurons in the hippocampus and in neurons in layers II and III of the cerebral cortex.^23^,^57^ Elevations in BDNF transcripts can occur within minutes to hours of initiation of vigorous exercise. BDNF production can also be regulated at the translational level in response to exercise and other stimuli, with levels of BDNF protein typically increasing by a greater percentage over baseline compared to the increase in BDNF mRNA levels.^58^ Compared to their more sedentary control counterparts, runner rats exhibit significantly more long-term potentiation (LTP) in response to theta rhythm-patterned stimulation, and this synaptic strengthening is associated with increased expression of the NR2B NMDA receptor subunit and BDNF.^44^

Activity-dependent production of nitric oxide may play an important role in the production of BDNF that occurs in response to exercise.^59^,^60^ Likely of particular importance for the effects of exercise on synaptic plasticity is the stimulation of BDNF protein production locally from BDNF mRNA associated with ribosomes in dendrites.^61^–^63^ In turn, BDNF induces the local (dendritic) translation of mRNAs encoding proteins critical for synaptic plasticity and learning and memory including Arc, *N*-methyl-d-aspartate (NMDA) receptor subunits, the postsynaptic density scaffolding protein Homer2, and CamKII.^64^,^65^ In neurogenic niches, such as that in the dentate gyrus of the hippocampus and the subventricular zone, BDNF produced by neurons acts upon neural progenitor cells to promote their differentiation into neurons and the survival of those newly generated neurons.^13^,^59^

BDNF is synthesized as a longer protein called proBDNF that has little or no ability to activate TrkB. Biologically active BDNF is generated by enzymatic cleavage of proBDNF; plasmin and matrix metalloprotease 9 are two proteases that have been shown to cleave proBDNF.^66^,^67^ Runner rats exhibit elevated activity of tissue-type plasminogen activator (tPA), an enzyme that converts plasminogen to plasmin, in the hippocampus, suggesting that exercise increases the generation of mature/active BDNF from proBDNF.^68^ The latter study further showed that administration of a tPA inhibitor negates the effects of exercise on hippocampal synaptic plasticity. Another study showed that the antidepressant effect of exercise is associated with elevated levels of mature BDNF and increased expression of tPA in the hippocampus.^69^

As laboratory rodents age, decrements in performance in learning and memory tasks occur that are associated with reduced LTP and decreased expression of BDNF in the dentate gyrus. These age-related deficits in hippocampal neuroplasticity can be ameliorated by exercise and environmental enrichment.^70^ In one study, the relative contributions of running and enrichment to the enhancement of neurogenesis in real world-like environments was determined by housing C57BL/6 mice under control, running, enrichment, or enrichment plus running conditions.^71^ Progenitor cell proliferation and differentiation into neurons, as well as BDNF expression, were increased significantly only when running wheels were available, suggesting that exercise may be more effective than intellectual challenges alone in stimulating BDNF production and neurogenesis.

Collectively, the available data suggest that exercise enhances synaptic plasticity by increasing activity in neuronal circuits related to the exercise (sensory association cortices, entorhinal cortex, and hippocampus, as well as motor output-related circuits). Glutamate, the major excitatory neurotransmitter in all of the aforementioned neuronal circuits, activates postsynaptic α-amino-3-hydroxy-5-methyl-4-isoxazolepropionic acid (AMPA) and NMDA receptors, resulting in local Ca^2+^ influx. Ca^2+^ then binds calmodulin to activate CaMKII, resulting in the activation of nitric oxide synthase and the transcription factor cAMP response element-binding (CREB).^72^ CREB then induces transcription of the *Bdnf* gene. In addition to glutamatergic signaling, activation of serotonergic and noradrenergic neurons, whose cell bodies are located in the brainstem, is necessary for a maximum effect of exercise on hippocampal BDNF production.^73^ BDNF mediates effects of exercise on synaptic plasticity and neurogenesis by activating TrkB. As evidence, ablation of TrkB in hippocampal neural progenitor cells impairs their proliferation and their ability to differentiate into neurons under basal conditions and abolishes exercise-induced neurogenesis.^74^ Downstream of TrkB, the PI3 kinase–Akt pathway plays a key role in effecting changes in synapses and neural progenitor cells. Thus, when a specific inhibitor of the PI3 kinase–Akt signaling pathway was infused into the brain, the ability of voluntary running to promote synaptic plasticity and the survival of newly generated neurons in the dentate gyrus of the hippocampus was compromised.^75^

Exercise has been shown to improve functional outcome in animal models of acute brain insults and progressive neurodegenerative disorders, and there is likely a role for BDNF signaling in the beneficial effects of exercise. For example, traumatic brain injury impairs cognitive function in rats, and postinjury exercise can enhance cognitive performance through a BDNF-mediated mechanism.^76^ Neurogenesis and maze learning were suppressed in a model of systemic infection/encephalitis, and moderate running restored the proliferation and neuronal differentiation of hippocampal neural progenitor cells and also reversed the maze learning deficit.^77^ In two different models of Alzheimer's disease (AD), running improved cognitive function, with notable reductions in markers of oxidative stress and inflammation in the brain.^78^,^79^ Mice and rats that are runners exhibit increased resistance of their dopaminergic neurons to degeneration and improved motor performance, in dopaminergic toxin-induced experimental Parkinson's disease (PD).^80^ In the latter study, it was found that levels of glial cell line–derived neurotrophic factor were greater in the striatum of runners compared to sedentary control animals. Exercise can also counteract adverse effects of a high-energy diet (a risk factor for stroke, and possibly AD and PD) on synaptic plasticity and cognitive function by a mechanism involving BDNF signaling.^81^

### DER and BDNF

Dietary energy restriction, either by limited daily feeding (sustained caloric restriction) or intermittent energy restriction (IER) (e.g., alternate day fasting), significantly extends health span and life span in rats and mice.^82^ DER can improve cognitive function and delay age-related cognitive impairment in rodents.^83^,^84^ Limited daily feeding enhances learning consolidation and synaptic plasticity by a mechanism involving increased expression of the NR2B subunit of the NMDA receptor.^85^ Recent findings have also demonstrated significant improvement in cognitive performance of nonhuman primates^86^ and humans^87^ maintained on caloric restriction diets. Hippocampal synapses in rats maintained on DER exhibit enhanced LTP.^88^ Neurogenesis is increased in mice maintained on an alternate-day fasting diet, by a mechanism involving BDNF signaling, and increased survival of newly generated neurons.^13^ However, whereas alternate day fasting increases expression of BDNF, limited daily feeding has less or no detectable effect on BDNF expression.^89^ Further work is therefore required to determine how different types of DER affect BDNF signaling in different regions of the nervous system, and if and how the changes in BDNF signaling mediate behavioral and metabolic responses to DER.

DER is remarkable in its ability to protect neurons against dysfunction and degeneration in experimental models of neurological disorders. For example, neuroprotective and/or disease-modifying effects of IER have been demonstrated in animal models of severe epileptic seizures,^90^ ischemic stroke,^91^,^92^ Huntington's disease,^12^,^90^ PD,^93^,^94^ and AD.^95^–^98^ Rats on a limited daily feeding (40% caloric restriction) diet exhibited recovery of spatial memory function, whereas rats fed *ad libitum* did not, in a model of cardiac arrest/global cerebral ischemia.^99^ Rats maintained for four months on a 30% caloric restriction diet and then subjected to traumatic brain injury exhibited reduced neuronal degeneration and superior performance in a spatial learning test, compared to brain injured rats on a control (*ad libitum*) diet.^100^ The improved outcome in the DER mice was associated with elevated levels of BDNF in the region of cerebral cortex around the site of injury and in the adjacent hippocampus. BDNF can protect neurons against oxidative, metabolic, and excitotoxic insults that are relevant to traumatic and ischemic brain injury by inducing the expression of antioxidant enzymes,^101^ anti-apoptotic Bcl-2 family members,^102^ and DNA repair enzymes (MPM, unpublished data).^103^

In a recent study, we maintained young, middle-aged, and old mice for three months on either IER (alternate day fasting) or *ad libitum* diets, and then subjected them to a focal ischemic stroke. The young and middle-aged mice in the IER group exhibited less brain damage and a better functional outcome, whereas IER had little or no protective effect in the old mice.^92^ BDNF, the protein chaperones HSP-70 and GRP-78, and the antioxidant enzyme HO-1 were upregulated by IER in the striatum and cortex of young and middle-aged, but not old mice. Moreover, the ability of IER to suppress the production of proinflammatory cytokines was attenuated in old mice.^92^ Thus, the ability of IER to enhance BDNF signaling and protect neurons against injury may be compromised with aging. The latter possibility leads to the conclusion that it is important to initiate DER (and exercise) in young adulthood or midlife to provide maximal protection of the brain against injury and disease late in life.

## BDNF mediates effects of energy intake on cognitive function

The roles of BDNF in synaptic plasticity, learning, and memory have been well studied and documented.^104^–^107^ BDNF has been shown to stimulate neurogenesis.^59^,^108^ The results of physiological studies also demonstrate that BDNF plays an important role in LTP (see Refs. 109 and 110 for reviews).

Epidemiological studies in human populations suggest that obesity and diabetes may increase risk for developing cognitive impairments and dementia.^111^–^113^ Multiple domains of cognitive function can be impaired in patients with diabetes.^114^ Several studies have provided evidence that diabetes is a risk factor for age-related cognitive impairment and AD.^115^,^116^ Studies with laboratory animal models have shown that overeating and obesity promote a range of major diseases including cardiovascular disease, diabetes, and many types of cancer.^117^ Moreover, higher energy intake induces metabolic disorders and, together with a sedentary lifestyle, may further add risk for development of cognitive impairment.^118^,^119^ A study conducted in rats indicated that animals maintained with food elevated in saturated fat or a diet with higher saturated fat and cholesterol committed memory errors compared to controls maintained with normal diets when they were tested in several different types of behavioral tasks.^120^,^121^

In a study from our lab using the triple-transgenic mouse model of AD (3xTgAD mice), the mice were maintained for one year (beginning at five months of age) on either *ad libitum* (control), 40% caloric restriction (CR), or IER (alternate-day fasting) diets. Behavioral testing of the 17-month-old 3xTgAD mice showed that those on the CR and IER diets exhibited higher levels of exploratory behavior and performed better in both the goal latency and probe trials of the water maze task compared to 3xTgAD mice on a control diet. 3xTgAD mice in the CR group also showed lower levels of Aβ1–40, Aβ1–42, and phospho-Tau in the hippocampus compared to the control diet group.^97^ At least one other study has reported that long-term IER can enhance cognitive performance in rats and mice.^85^

### Synaptic plasticity and BDNF

The mechanism(s) by which BDNF enables and enforces the changes in synaptic structure and function believed to underlie learning and memory are being elucidated. Although studies have shown that energy restriction affects BDNF expression in multiple brain areas, including cortical and subcortical regions, the hippocampus has received the most attention in various studies. The hippocampus has been a focus for studies of cognition-related synaptic plasticity because of the layered structural organization of the neurons and synaptic connections that provides the opportunity to readily perform electrophysiological recordings of synaptic transmission and long-term changes in synaptic strength. In addition, many of the currently well-characterized behavioral tasks used to evaluate memory, particularly for rodents, are hippocampus dependent. BDNF and LTP have received a great deal of attention in neuroscience research (see Refs. 109 and 110 for reviews). BDNF plays a critical role in activity-induced expression of proteins and in generating sustained structural and functional changes at hippocampal synapses. In particular, BDNF is sufficient to induce the transformation of early to late-phase LTP.^109^ One of our recent studies showed that rats maintained on a high-fat, high-glucose diet exhibited impaired spatial learning ability, reduced hippocampal dendritic spine density, and reduced LTP at Schaffer collateral–CA1 synapses.^122^ These changes were associated with reductions in levels of BDNF in the hippocampus. Therefore, a diabetogenic high-calorie diet reduces hippocampal synaptic plasticity and impairs cognitive function, possibly by impairing BDNF-mediated effects on dendritic spines. In a mouse model, we also found that excessive energy intake resulted in impaired leptin signaling and hippocampal neurogenesis, and was associated with deficits in synaptic plasticity at the perforant path—dentate granule neuron synapses and impaired hippocampus-dependent memory.^45^ Other studies reported a complementary result showing that a CR protocol prevents rats from developing age-related deficits in LTP and sustains NMDA and AMPA glutamate receptor levels in the hippocampus.^88^,^123^

### BDNF and cellular energy metabolism

An additional mechanism by which BDNF may mediate beneficial effects of DER on cognition involves enhancing cellular energy metabolism.^124^ In a recent study, it was shown that when 3xTgAD mice were fed a diet supplemented with 2-deoxyglucose (2DG) for seven weeks, levels of Aβ in their brains decreased, levels of BDNF increased, and neuronal bioenergetic capacity was increased.^125^ Mice on the 2DG diet also exhibited elevated levels of ketone bodies, which are fatty acids known to affect neuronal excitability and to protect neurons against excitotoxic and metabolic insults.^126^ BDNF has been shown to directly modify neuronal energy metabolism. For example, BDNF induces the expression of the monocarboxylate transporter that enables the use of lactate as an alternative energy source.^127^ The ability of BDNF to induce the monocarboxylate transporter was blocked by inhibitors of PI3 kinase and p42/p44 MAP kinases. Another study showed that BDNF can stimulate glucose utilization and the upregulation of the glucose transporter, GLUT3 in cultured neurons.^128^ BDNF also stimulates sodium-dependent amino acid transport and increases protein synthesis. The abilities of BDNF to increase cellular energy availability and enhance protein synthesis may play important roles in synaptic plasticity because energy substrates (ATP and NAD^+^) and protein synthesis are critical for synaptic plasticity and learning and memory.^129^,^130^

## The big picture: BDNF as an integrator of behavioral and neuroendocrine regulation of energy metabolism

**Figure 4:**
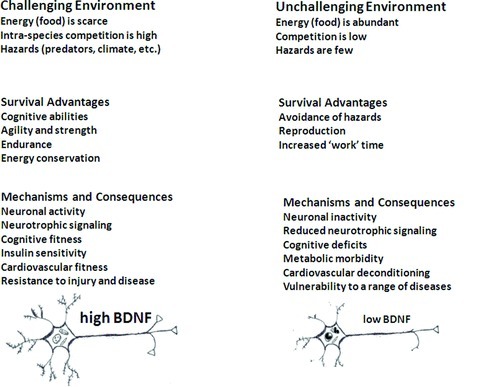
Characteristics of individuals living in natural or self-imposed environments at the two ends of the energy spectrum. (Left) An environment where food is scarce and competition and hazards are high poses major physical and cognitive challenges. From an evolutionary perspective, survival is favored by phenotypes that include superior cognitive abilities, strength and agility, endurance, and the ability to conserve energy. In this setting, neurons are active, and the production of neurotrophic factors such as BDNF are elevated. Emerging evidence suggests that BDNF mediates multiple responses of the individual to a Spartan environment, including enhanced neuronal plasticity, increased insulin sensitivity, improved cardiovascular fitness, and resistance of tissues to injury and disease. The different BDNF-mediated responses to a challenging environment can be considered a coordinated, adaptive cellular- and systems-level response to the reduced energy availability and increased energy expenditure required to survive. (Right) In an environment where food is abundant and the need for energy expenditure (exercise) minimal, individuals can increase their attention to reproduction and their occupation within the society. This environment results in reduced activation of adaptive stress response pathways, and thereby reduced BDNF signaling, insulin resistance, cardiovascular deconditioning, and susceptibility to a range of diseases, particularly age-related diseases.

Consider mammals, including our primate predecessors and ourselves, humans, in the natural environments in which they evolved. Further, consider two very different ends of the energy equation with regards to the availability/accessibility of energy (food) resources within the environment (Fig. 4). In one scenario (challenging environment), food is scarce, competition within and among species for the energy is high, and hazards such as predation also enter into the energy equation on both the acquisition and expenditure sides. Such challenging environments demand relatively higher levels of processing and retention (memory) of information from the environment (particularly sights, sounds, and odors). The underlying activity in neuronal circuits involves BDNF production and signaling, which promotes synaptic plasticity, the growth of dendrites, and neurogenesis. Individuals in physically challenging environments are at increased risk of injury, including damage to the nervous system. BDNF may provide a survival advantage to those suffering trauma to the nervous system by stimulating mitochondrial biogenesis and resistance of neurons to injury by upregulating the expression of genes encoding cytoprotective proteins. Recent findings suggest that BDNF signaling in the brain mediates the positive effects of a cognitively and physically challenging environment on energy metabolism including increased insulin sensitivity,^17^ increased genesis of brown fat,^131^,^132^ and cardiovascular fitness.^133^

In the second scenario (unchallenging environment), energy is readily available and there are few hazards to obtaining the energy (Fig. 4). The time that would otherwise be used for food acquisition is used for reproduction and work to obtain other types of resources that provide an advantage to the individuals and their families. Because energy intake is increased and physical activity reduced in such an environment, BDNF signaling is reduced in neurons in the brain. Reduced BDNF signaling enhances food intake, reduces insulin sensitivity, deconditions the cardiovascular system, reduces cognitive abilities, and increases vulnerability to age-related diseases.

In summary, individuals will be lean, alert, and cognitively sharp when living in an environment in which there are major challenges for energy acquisition. This same (healthy) physical and cognitive phenotype can be achieved by self-imposed energy restriction and exercise routines in humans. BDNF signaling plays major roles in mediating adaptive responses of the nervous, cardiovascular, and energy-regulating organ systems in response to exercise and energy restriction. Efforts to develop pharmacological and molecular genetic methods for activating BDNF-signaling pathways may result in novel therapeutic treatments for a range of metabolic and neurological disorders.
